# Chronic Myelomonocytic Leukemia-Associated Immune Thrombocytopenic Purpura: A Report of a Rare Case and a Review of Literature

**DOI:** 10.7759/cureus.55904

**Published:** 2024-03-10

**Authors:** Ghadir M Nasreddine, Solay Farhat, Zeinab M Hammoud, Firas Saad, Wajih Saad

**Affiliations:** 1 Department of Hematology and Oncology, Lebanese University, Faculty of Medical Sciences, Beirut, LBN; 2 Department of Hematology and Oncology, Morristown Medical Center, New Jersey, USA; 3 Department of Hematology and Oncology, Al-Zahraa Hospital University Medical Center, Beirut, LBN

**Keywords:** azacitidine revolade, case report, crohn’s disease, immune thrombocytopenic purpura, chronic myelomonocytic leukemia

## Abstract

Chronic myelomonocytic leukemia (CMML) presents as a complex hematologic malignancy with myelodysplastic and myeloproliferative features. Our case report explores the rare coexistence of CMML with immune thrombocytopenic purpura (ITP) in a 63-year-old female patient. CMML diagnosis followed World Health Organization criteria, and the patient was classified as having high-risk myelodysplastic syndrome (MDS)-CMML stage 2. Initial treatment with subcutaneous azacytidine for CMML proved partially effective, highlighting persistent severe thrombocytopenia. Subsequent investigations revealed secondary ITP associated with Crohn's disease. Conventional ITP therapies, including high-dose steroids and intravenous immunoglobulin, showed limited efficacy. Eltrombopag, a thrombopoietin receptor agonist, was initiated, resulting in the normalization of platelet counts within six weeks. Our case emphasizes the diagnostic challenges and intricate treatment landscape of CMML-associated ITP, suggesting eltrombopag as a potential therapeutic option in refractory cases. The study contributes to the evolving understanding of the complex interplay between myeloid disorders and immune-mediated hematological conditions, calling for personalized and multidisciplinary approaches to enhance patient outcomes.

## Introduction

In the intricate landscape of hematologic disorders, chronic myelomonocytic leukemia (CMML) emerges as a multifaceted entity, intertwining its pathophysiology with immune thrombocytopenic purpura (ITP). CMML emerges as a rare, heterogeneous, and complex hematologic malignancy, embodying both myelodysplastic syndrome (MDS) and myeloproliferative neoplasm (MPN) features within the spectrum of myeloid disorders. This distinctive entity is marked by clonal expansion of hematopoietic stem cells, resulting in sustained monocytosis, possible bone marrow lineage dysplasia, and an inherent risk of progression to acute myeloid leukemia (AML) (15-30% within 3 to 5 years) [1]. Notably, CMML primarily manifests in elderly patients aged between 65 and 75 years, displaying a male predominance [2]. The condition presents through a spectrum of nonspecific clinical features due to cytopenia in dysplastic CMML such as fatigue caused by anemia, increased susceptibility to infections from neutropenia, and a propensity for bruising due to thrombocytopenia. Other constitutional symptoms (fever, weight loss, night sweats, pruritus) are present in proliferative CMML [2]. Depending on the leukocyte count, CMML is subclassified into the dysplastic variant (MDS-CMML) if this count is ≤13x10^9^/L and the proliferative variant (MPN-CMML) if the count is >13x10^9^/L. The diagnosis of CMML is made using the following criteria according to the 2016 World Health Organization classification: (i) sustained monocytosis (>3 months) with an absolute monocyte count (AMC) >500/μL; (ii) exclusion of other diseases that could cause monocytosis such as CML/BCR-ABL1+ and other MPNs; and (iii) blast count <20% in peripheral blood and bone marrow. Also, ≥1 bone marrow myeloid lineage dysplasia must be present. If dysplasia is not found, clonal cytogenetic or molecular abnormalities for CMML and/or the detection of CMML by flow cytometry and immunohistochemistry should make the diagnosis [3].

Staging of CMML is based on the percentage of blasts in the peripheral blood and bone marrow. CMML is considered stage 1 if blasts are <4% in peripheral blood and <9% in the bone marrow; however, CMML-2 is diagnosed if blasts are >4% in peripheral blood and 10-19% in the bone marrow. Finally, stage 0 (CMML-0) is diagnosed if blasts are <2% in peripheral blood and <5% in bone marrow. Auer rods could be present only in CMML-2. Treatment of CMML differs between moderate to high-risk groups (≥1: MPN-CMML, blast >5%, transfusion-dependent patient, and cytogenetic abnormalities) and lower-risk groups. Allogeneic hematopoietic stem cell transplantation (HSCT) is considered the only curative option for patients with CMML, especially in high-risk and fit patients. For patients not suitable for transplantation, or those with lower risk and symptomatic, hypomethylating agents (e.g., azacytidine) and hydroxyurea are considered. In asymptomatic lower-risk groups, the choice between observation and treatment is individualized [4].

ITP mainly presents as an autoimmune condition targeting platelets, causing their premature destruction and resulting in thrombocytopenia. Consequently, individuals may experience signs and symptoms of bleeding ranging from mild to severe. The diagnosis is made after excluding other causes of thrombocytopenia [5]. Bone marrow accelerates the production of platelets to compensate for their destruction. Thus, the presence of megakaryocytic hyperplasia and other lineages is normal on bone marrow aspiration [6].

Though uncommon, the link between CMML and ITP has been reported in clinical literature. Indeed, their concurrent presence poses diagnostic and therapeutic challenges, as the management of one condition may influence the course of the other. Understanding the underlying mechanisms linking these disorders is crucial for effective patient management and underscores the need for personalized and multidisciplinary approaches.

In this case report, we present a detailed examination of a patient diagnosed with both CMML and ITP, shedding light on the complex relationship between these disorders. Through a comprehensive analysis of clinical, hematological, and molecular data, we aim to contribute to the growing body of knowledge surrounding the coexistence of CMML and ITP, providing insights that may inform future diagnostic and therapeutic strategies for patients facing this intricate hematological conundrum. Written informed consent was obtained from the patient for the publication of any potentially identifiable images or data included in this article.

## Case presentation

**Figure 1:**
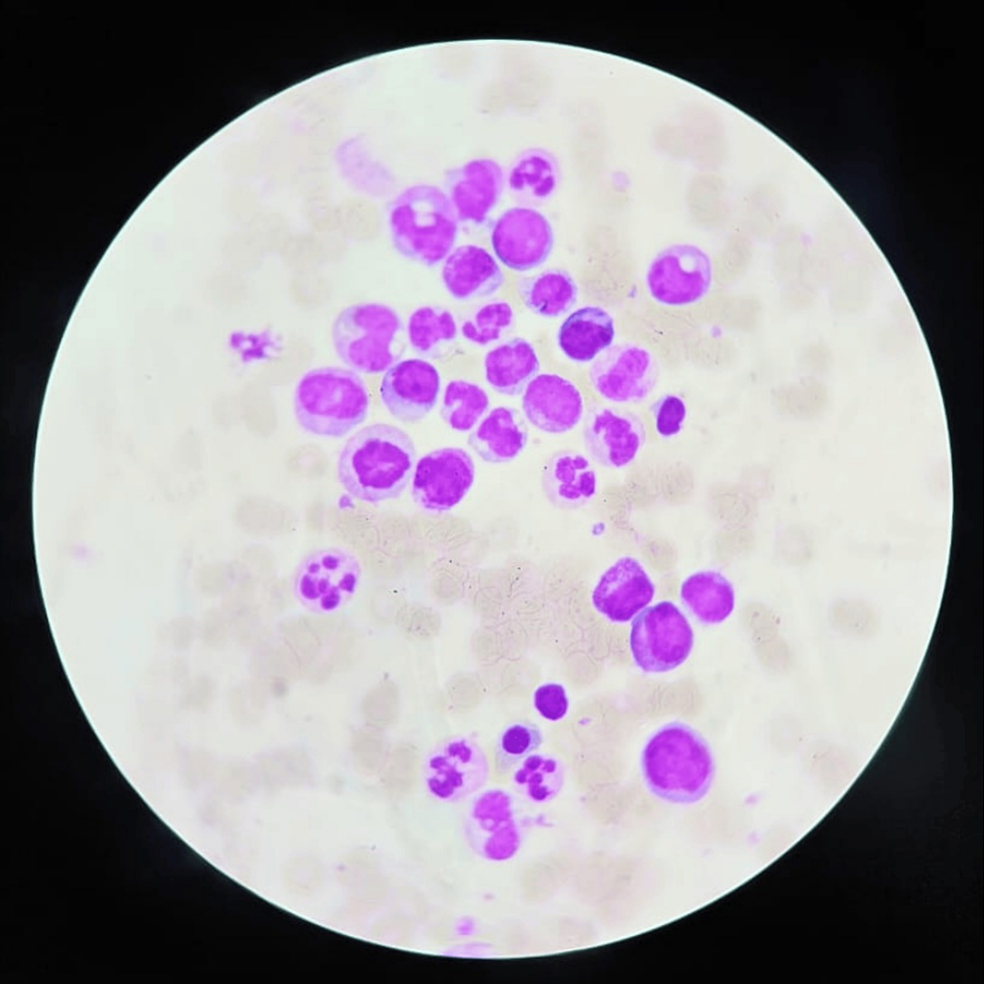
Monoblast in the bone marrow aspirate smear Large cells with abundant cytoplasm and round nuclei with one or more nucleoli are seen.

This case report describes a 63-year-old, female, heavy smoker with a medical history of type 2 diabetes mellitus, dyslipidemia, hypothyroidism (post-thyroidectomy), and osteoporosis. She presented with a one-month history of intermittent cramping abdominal pain and loose stools. A colonoscopy revealed a single ulcer in the terminal ileum, compatible with Crohn's disease on histopathology. Additionally, she exhibited leukocytosis (22,000/μL), macrocytic anemia (Hb=7.2g/dl, mean corpuscular volume (MCV)=105), thrombocytopenia (33,000/μL), and monocytosis (18%) with an absolute monocyte count (AMC) of 3660/μL, neutrophil 42%, and absolute neutrophil count (ANC) of 10,990/μL with elevated lactate dehydrogenase (LDH) of 730 and erythrocyte sedimentation rate (ESR) of 145. She was admitted to our hospital for further investigations. Upon presentation, her vital signs were stable. She complained only of fatigue and denied weight loss, fever, and night sweats. On physical examination, she exhibited pallor, pale conjunctivae, no palpable lymph nodes, no hepatosplenomegaly, and a soft abdomen with positive bowel sounds and no petechiae or ecchymosis. Peripheral smear revealed leukocytosis, immature monocytes and promonocytes, myelocytes and promyelocytes, rare blast cells and Auer rods, and severe thrombocytopenia. No hepatosplenomegaly was seen on echo abdomen pelvis. Bone marrow aspirate showed hypercellularity, trilineage dysplasia, monocytosis (34%), and increased blasts (14%) on flow cytometry as shown in Figure 1, and findings were compatible with chronic myelomonocytic leukemia using World Health Organization 5th edition (WHO5) criteria. Molecular analysis showed no detectable translocations, including the common t(9,22)(BCR-ABL1), and the karyotype was normal. CMML was classified as high-risk MDS-CMML stage 2 (CMML-2) due to the presence of trilineage dysplasia regardless of leukocytosis.

After diagnosis, the patient started on subcutaneous azacytidine, a hypomethylating agent, for seven days as the specific treatment for CMML. However, despite treatment, the patient experienced persistent severe thrombocytopenia with platelet counts remaining below 5000/ μL, which failed to resolve on daily irradiated platelet transfusion. Therefore, other causes of thrombocytopenia were excluded (negative viral serology, no other infectious causes, no disseminated intravascular coagulopathy, no drug-induced thrombocytopenia, and normal vitamin B12/folate levels). Thus, after one week, reassessment of bone marrow aspirate demonstrated notable hypercellularity with trilineage hematopoiesis with dysplasia, erythropoiesis, and granulopoiesis increased, megakaryocytes present with occasional dysplastic features, a monocyte count of 16%, and a reduction in blast cells to 4%, suggestive of a favorable response to therapy and excluding a central cause of thrombocytopenia. Given the recent diagnosis of Crohn's disease, an autoimmune condition characterized by inflammation and tissue damage in the intestinal tract [7] and secondary ITP associated with autoimmune diseases was considered. The presence of autoimmune disease was supported by positive findings in the antinuclear antibody (ANA) panel for anti-RO52 and AMA-M2. Although anti-platelet antibodies were negative, their negativity does not rule out the presence of ITP due to their low sensitivity [8].

Subsequently, the patient underwent a course of high-dose steroid therapy (dexamethasone 8mg IV q8hr), which failed to alleviate the thrombocytopenia. Following this, intravenous immunoglobulin (IVIg) at a dosage of 60g was administered without success. Finally, the patient was initiated on an oral thrombopoietin receptor agonist agent (eltrombopag/revolade) 50 mg daily, and the dose was increased by 25 mg after 1 week due to persistent thrombocytopenia. The patient took the second cycle of azacitidine on day 28. After seven days, bone marrow aspirate was repeated for reevaluation before discharge, and the result was satisfactory with trilineage dysplasia, maturing granulocytes and erythrocytes, presence of megakaryocytes with occasional dysplastic features, a monocyte count of 5%, and blasts decreased to <1%. Also, complete blood count after the second cycle of azacitidine showed resolution of leukocytosis and monocytosis (WBC=4.56 x 10³/μL, monocyte=5%, AMC=260/μL), Hb=9, and a slight increase in platelet count to 9000/μL after three weeks of therapy with eltrombopag. Finally, the patient was discharged on Revolade, and the platelet count normalized three weeks later.

## Discussion

As previously mentioned, CMML is characterized by abnormal growth of myeloid cells causing monocytosis, with features of both myelodysplastic and myeloproliferative disorders, and poses diagnostic and therapeutic challenges on its own. Therefore, the co-occurrence of CMML with ITP, autoimmune-mediated platelet destruction, creates a distinctive clinical scenario and increases disease complexity. Thus, understanding the characteristics and management of this occurrence in affected individuals is essential. Our case study, complemented by a comprehensive review of the current literature, provides insights into the diagnostic and therapeutic complexities arising from the intersection of these two hematological conditions. Only 10-20% of cases involving MDS or CMML are linked to various autoimmune diseases, with the association with ITP being particularly rare [9]. This underscores the importance of recognizing the potential association between ITP and myeloid neoplasms such as CMML. In a study conducted by Jachiet V et al., spanning 16 French centers from January 1999 to July 2019, they observed only 77 patients with MDS/CMML and ITP, of which 41 were included [9]. Among them, ITP was diagnosed simultaneously with MDS/CMML in 17 patients (41%) while it occurred before MDS/CMML in 16 patients (39%), and manifested after MDS/CMML in 8 (20%) patients [9]. Additionally, another study by Hadjadj J et al. confirmed that this association is rare, mentioning only 21 cases of ITP associated with CMML in the literature [10]. Indeed, ITP in CMML patients exhibits distinct characteristics beyond typical ITP manifestations, often associated with an increased disease risk and more severe bleeding compared to primary ITP. This is evident because thrombocytopenia worsens when ITP coexists with MDS/CMML due to dysmegakaryopoiesis and platelet dysfunction [9]. Moreover, patients with associated diseases generally experience better leukemia-free survival compared to those with CMML without ITP. To note, primary ITP is characterized by a more favorable prognosis, including a higher likelihood of spontaneous remission and a reduced need for treatment [9].

The precise underlying reasons for the correlation between autoimmune disorders and CMML remain elusive, primarily due to limited available data resulting from a lack of comprehensive large-scale systemic reviews of clinicopathological findings regarding this association with autoimmune phenomena. However, a study conducted by Peker D et al. in 2015, within a single institution, conduct into potential explanations for this co-occurrence [11]. First, shared genetic factors may heighten susceptibility to both CMML and autoimmune disorders. Second, CMML patients often exhibit elevated levels of inflammatory cytokines, which can activate the immune system and potentially contribute to autoimmune reactions similar to those observed in ITP. Overall, this study underscores the intricate interplay between CMML and autoimmune cytopenias, suggesting multiple contributing factors. Further research is imperative to elucidate the exact mechanisms involved and to enhance diagnostic and therapeutic strategies for affected individuals [11].

Diagnosing CMML associated with ITP involves a comprehensive approach, incorporating clinical assessments, laboratory tests such as a complete blood count and examination of peripheral blood smears, and meticulous analysis of bone marrow. This comprehensive diagnostic approach is essential for gaining insights into the intricate interplay between CMML and ITP, given the varied clinical scenarios observed in this uncommon coexistence. Significantly, understanding the chronological sequence of diagnoses offers valuable perspectives on the dynamics of these hematological disorders when they manifest concurrently. Distinguishing immune-related peripheral thrombocytopenia from centrally derived thrombocytopenia, such as that caused by bone marrow failure, becomes particularly intricate within the context of MDS/CMML [9]. This challenge contributes to the risk of misdiagnosis and underscores the importance of precision in understanding the origin of thrombocytopenia. Furthermore, the overlap with other underlying conditions adds a layer of difficulty to the diagnostic process. Patients with ITP may harbor concurrent conditions, such as von Willebrand disease or primitive immunologic abnormalities, which can mimic or coexist with ITP, leading to potential misdiagnoses [12]. Additionally, the heterogeneous presentation of ITP in the context of CMML introduces another dimension of difficulty. Notably, this variant may manifest with a more severe bleeding profile and show refractoriness to first-line treatments such as corticosteroids when compared to primary ITP [9,13]. In our case, the definitive diagnosis of ITP in a patient already diagnosed with CMML was done by comprehensive exclusion of all possible other causes of the presented thrombocytopenia. Notably, the patient didn’t present with any bruising or any visible sources of bleeding, with a negative fecal occult blood (FOB) test indicating the absence of any internal microscopic bleeding. Thus, the severely low platelets count (2 x 10^3^/ul) even post-platelets transfusion and steroid therapy couldn’t be attributed to any possible bleeding. To evaluate other aspects of blood clotting, a normal INR indicated an absence of any other abnormality in the blood clotting process. Additionally, infectious causes were ruled out, and drug-induced thrombocytopenia was not observed. Finally, megakaryocytes on bone marrow smear indicated peripheral platelet destruction and positive antibodies in the ANA panel support an autoimmune mechanism underlying the patient's ITP.

Hadjadj et al. indicated that the recommended approach for treating CMML-associated ITP should align with existing guidelines for primary ITP [10]. First-line interventions typically involve first-line therapies, such as high-dose dexamethasone or prednisone, along with IVIg, either administered individually or in combination [9,14]. However, a recent study published in 2021 by Jachiet V et al. demonstrated that patients with MDS/CMML-associated ITP have shown a lower response rate to steroids or IVIg, and experienced more frequent relapses after first-line therapy when compared to patients with primary ITP [9]. Hence, this suggests that addressing ITP in CMML patients might demand special attention owing to the distinctive features and clinical progression of ITP within the context of CMML. It is crucial to implement personalized treatment strategies and adhere to current ITP management guidelines to enhance patient outcomes. The management of CMML-associated ITP poses significant challenges, particularly as the majority of cases prove refractory to conventional treatments, leading to a limited array of therapeutic options. In contrast to typical approaches employed for primary ITP, which often include corticosteroids and IVIg as standard treatments, these may not be effective for patients with CMML-associated ITP [15]. This was also observed in our case, where the patient showed no response to high-dose dexamethasone and IVIg.

In the pursuit of alternative therapeutic avenues, eltrombopag, a thrombopoietin receptor agonist, has emerged as a reported effective intervention for managing refractory thrombocytopenia in CMML patients [10,15]. This development signifies a potential breakthrough in addressing the unique challenges posed by CMML-associated ITP, offering a promising option in cases where traditional treatments fall short. A study by Gao Y et al.reported successful eltrombopag treatment in patients with CMML-associated ITP [15]. The recommended eltrombopag dosage for CMML-associated ITP entails an initial daily dose of 50 mg for adults. In cases where a significant increase in platelet counts is not observed within two to three weeks, a dosage escalation may be considered. Adjustments to the dose are further influenced by platelet counts, including a potential increase of 25 mg if counts fall below 50 x 10^9^/L or a decrease if counts reach the range of 200 to 400 x 10^9^/L at any point [16]. In addition, clinicians should take caution regarding the potential risk of acute leukemia transformation associated with the use of thrombopoietin receptor agonists in the context of MDS/CMML [9]. In our case, the patient initiated eltrombopag therapy and achieved normalization of platelet counts within six weeks. However, the intricate nature of CMML and its interaction with ITP emphasizes the ongoing necessity for further research to refine and broaden treatment approaches for this complex hematological condition.

## Conclusions

In conclusion, the coexistence of CMML with ITP underscores the intricate overlap between myeloid disorders and immune-mediated hematological conditions. The challenges in diagnosis and management highlighted by this rarity and diverse clinical presentations emphasize the complexity of such cases. Our case study contributes valuable insights into the diagnostic and therapeutic complexities arising from this convergence. The distinctive features observed, such as refractory thrombocytopenia, necessitate a comprehensive diagnostic approach, including various assessments and imaging studies. As treatment complexities persist, ongoing research is crucial to refine modalities and improve patient outcomes in the evolving landscape of CMML-associated ITP.
